# Patients’ Values and Desire for Autonomy: An Empirical Study from Poland

**DOI:** 10.1007/s11673-023-10241-y

**Published:** 2023-03-24

**Authors:** Agnieszka Olchowska-Kotala, Agata Strządała, Jarosław Barański

**Affiliations:** Department of Medical Humanities and Social Sciences, Wroclaw Medical University, ul Mikulicza-Radeckiego 7, 50-368 Wroclaw, Poland

**Keywords:** Desire for Autonomy, Healthcare Decision, Schwartz’s Theory, Value

## Abstract

There is a growing body of literature concerning factors that can influence patients’ perception, preferences, or expectations with regard to autonomy in making healthcare decisions. Although many factors responsible for the desire for autonomy in medical decision-making are already recognized, little is known about how the desire for autonomy is related to values, which refer to important goals of human actions. The present study was designed to determine the relationship between the desire for autonomy and basic personal values drawn on Schwartz’s value theory. We conducted survey in two age groups: younger and older adults. In the group of younger adults, the desire for autonomy was associated with the need to be appreciated as a person, motivation to act independently, and the abandonment of traditional order and values, whereas in the group of older adults, with independent thinking and a lack of humility. Our study highlighted that the desire for autonomy may result from slightly different reasons in people of particular age groups. These findings might be helpful for medical professionals in developing accurate communication patterns with different age groups of patients.

## Introduction

The principle of autonomy, which in the second half of the twentieth century became the cornerstone of medical ethics, has changed the perception of the doctor–patient relationship and has raised the importance of making decisions in medical practice. What is more, the iconic ethical codes such as the Nuremberg Code (1947, British Medical Journal, 1996), Declaration of Helsinki (1964), and Belmont Report (1978) proclaimed informed consent (and informed refusal) as an essential condition of the research on human subjects. Informed consent in medical research paved the way for respecting patients’ autonomy in medical treatment. Respecting patients’ autonomy is one of the newly added ethical duties of medical professionals according to the Declaration of Geneva (World Medical Association, Anon, 2017).

In the medical context, autonomy is most often defined as self-determination expressed by making voluntary and informed decisions by a competent person (Beauchamp and Childress 2019). On the other hand, patients’ autonomy is a complex and multi-dimensional construct. The notion of individual autonomy and its relation to informed consent remain a matter of dispute and have been questioned by feminist criticism (Lee 2007), Confucian (Yu and Li 2014), and African bioethics (Onuoha 2007; Mfutso-Bengo and Masiye 2011), etc. Not only are there many competing conceptions of autonomy (i.e., individualistic, relational, Kantian, utilitarian, Dworkin’s), but also different ways of implementing it into medical practice (paternalistic, informative, shared decision-making) (Varelius 2006; Dworkin 2015; Entwistle et al. 2010).

The question of what levels of autonomy in decision-making processes are actually expected by different groups of patients is still open and often debated. Moreover, a patient’s autonomy is understood differently across cultures, e.g., with regard to physicians’ willingness to inform patients about their diagnoses and prognoses. For instance, according to studies on informing oncological patients about their unfavourable diagnoses and prognoses, Polish physicians seem to be more resistant to revealing the whole truth to their patients—only 24 per cent claimed that they would always tell their patients the entire truth as compared to 81 per cent of Norwegians physicians (Leppert, Majkowicz, and Forycka 2013; Loge et al. 1996). Currently, under the Polish law and the Polish Medical Code of Ethics, physicians are exempt from informing patients about their condition only if clearly requested to do so by the patient (*The Polish Medical Code of Ethics. Announcement No. 1/04/IV of the President of the Supreme Medical Council,* Naczelna Izba Lekarska, 2004) (Chapter 1, Art. 16, item 1). Moreover, physicians are required to take into account patients’ will in the decision-making process (*The Polish Medical Code of Ethics. Announcement No. 1/04/IV of the President of the Supreme Medical Council*, Naczelna Izba Lekarska, 2004) (Chapter 1, Art. 13, 15). In this context, patients’ preferences of whether to be informed or not are of crucial importance.

There is a growing body of literature concerning factors that can influence patients’ perceptions, preferences, or expectations with regard to autonomy. Seeking autonomy can be influenced by health status (acute vs. chronic diseases) (Levinson et al. 2005; Chewning et al. 2012), the level of satisfaction with healthcare and trust in physicians (Lee and Lin 2010), culture (e.g., the level of individualism) (Veatch 1997; Tao and Po-Wah 2002), and individual differences (e.g., personality traits) (Flynn and Smith 2007). Studies indicate that people have different preferences concerning their own involvement in the treatment process. Some prefer to have only partial control over this process and they do not want to be principal decision-makers. In general, young, educated, single, healthy people (Ende et al. 1989; Levinson et al. 2005) women and people recently involved in decision-making processes show a greater desire for autonomy (Cullati et al. 2011).

**Figure 1.**
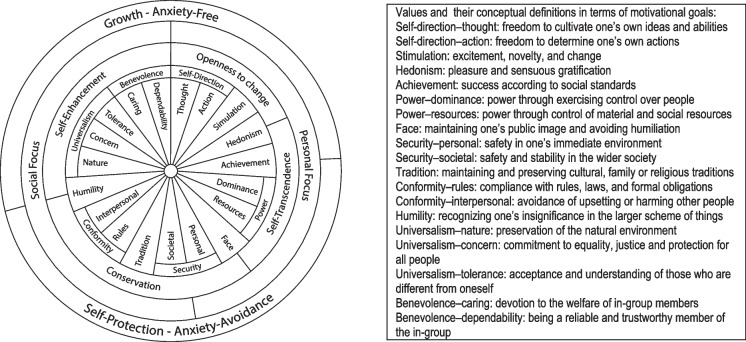
Circular motivational continuum of 19 values with sources that underlie their order. (adapted from Schwartz et al. 2012).

Although many factors responsible for the desire for autonomy in medical decision-making are already recognized, little is known about how the desire for autonomy is related to values, which refer to important goals of human actions. Considering informed consent as a form of enforcing the patient’s autonomy in everyday medical care, we focused on its fundamental components, such as informative and volitional. The informative aspect is related to the need for information, while the volitional one is related to independent decision-making. This perspective was adopted because we were most interested in the communicative aspect of the physician–patient relationship and why some group of patients wants to be informed and actively participate in decision-making, while others do not. The results could shed light on significant differences between patients in terms of their communication needs*.* Therefore, we decided to address this issue by adopting Schwartz’s value theory (Schwartz 2012; Schwartz et al. 2012), which explains peoples’ motivation to take action. According to this theory, people’s basic values are beliefs, representing desirable, abstract goals that motivate behaviour by serving as standards according to which we behave and evaluate actions, other people, and events. In other words, values are desirable, trans-situational goals that vary in their importance as guiding principles in people’s lives (Schwartz 1994). Value theory postulates that people seek to express their important values in behaviour mainly for two reasons: in order to attain the goals that are important to them and to affirm the values that are central to their self-identities. Schwartz’s refined theory distinguishes a set of nineteen values, which are presented in the form of a circle representing a motivational continuum (values located on the one side of the circle are to some extent compatible with each other, while those on the opposite side are contradictory) (Schwartz et al. 2012). The pattern of relations of conflict and compatibility among values is represented in the structure in Figure 1. The conflict of values results from conflicting needs, e.g., Security, Conformity, and Tradition are in opposition to Stimulation, Self-direction, and, to some extent, to Hedonism. According to Schwartz, pursuing one type of values will always conflict with other types of values on the opposite side of the circle. Values that share compatible motivational goals correlate positively and are close to one another in the circular arrangement. Values that express conflicting motivational goals correlate less positively, or even negatively (Roccas et al. 2002). Schwartz states that values are universal, i.e., they can be recognized across societies. However, the importance of values differs among cultures and individuals.

The Schwartz value theory has proved to be a powerful tool used in many studies on various aspects of life and values were found systematically related to daily behaviours as well as to major life choices (Schwartz et al. 2017; Sagiv, Sverdlik, and Schwarz 2011) including medicine. For instance, Cataldi et al. (2019) studied the relevance of people’s values to their attitudes to vaccination. Authors found the relationship between positive attitudes to vaccination and two subscales: Conformity and Universalism, whereas Self-direction was associated with late vaccination.

Due to the empirical evidence of the relationship between values and people’s specific behaviours and decisions (Skimina et al. 2019), we decided to analyse the usefulness of Schwartz’s theory for predicting the level of patients’ desire for autonomy. To the best of our knowledge, this has not yet been investigated in the literature. An important premise of this research is that previous studies based on Schwartz’s values taxonomy have shown the significant role age plays in shaping individual values (Robinson 2013). This phenomenon has been confirmed by studies of Western and non-Western cultures (Marcus, Ceylan, and Ergin 2017). Moreover, in the study of thirteen European countries, Robinson found that values are positively correlated with age (Robinson 2013). Typically, older people tend to be more conservative and attached to tradition but less prone to self-enhancing activities such as searching for new stimulation or change. Younger people are more open to self-improvement and change but less conservative. These general differences between age groups are the basis for analysing the relationship between the desire for autonomy and values in relation to a specific age group.

As we have already mentioned, there are many different ways of understanding autonomy. In our study, similarly to other authors (Cullati et al. 2011), we treated autonomy as the patients’ desire for information and their willingness to make medical decisions (we have not considered other elements of a patient’s autonomy, e.g., the right to privacy and confidentiality). The primary goal of the current study was to assess the utility of 19 values for predicting the desire for autonomy (DA). Previous studies confirmed that DA was related to the age of the study participants, i.e., the younger the person, the stronger the DA (Ende et al. 1989; Levinson et al. 2005). However, the relationship between DA and the structure of values has not been examined. In this study, we wanted to investigate whether DA is motivated by the same factors among younger people as it is among older people.

As a preliminary step, we wanted to check whether young adults in our sample, like those in other nations’ studies, had a stronger desire for autonomy than older adults, so we tested the hypothesis: The desire for autonomy in medical decision-making is higher among younger adults as compared to older adults (Hypothesis 1). Concerning values, since autonomy is defined as the capacity to think, decide, and act freely and independently, we presumed that people who valued Self-direction in their thinking and actions would have a higher DA. Therefore, we formulated the following hypothesis: Participants from both groups who attribute high importance to Self-direction (thought and action) have higher DA (Hypothesis 2). In terms of the relationship between DA and the other Schwartz values, we adopted an exploratory approach and did not formulate hypotheses.

## Methods

### Participants

The survey was conducted in southern Poland with a convenience sample. A total of 421 respondents participated in the study. The study was conducted in two age groups. The first group consisted of students from Wroclaw (n = 222; 151 women and 71 men) aged 18–27 years (*M*_*age*_=20.5; *SD* =1.61). The second group consisted of older adults recruited by students among their friends and families based on the following eligibility criteria: (1) age ≥40 years, (b) literacy (individuals with signal difficulties in reading or interpreting questions were excluded), and (c) lack of any medical condition that would affect their ability to participate. Students were trained in the proper selection of participants, and they handed out and collected questionnaires among older adults. The study was conducted in November 2020. Out of 215 respondents who agreed to participate, sixteen were excluded from the analyses, because they did not completely fill out the questionnaires. In total, 199 people were enrolled. This group comprised of 123 women and seventy-six men aged 40–90 years (*M*_*age*_=62.7; *SD*=11.72). The educational level in this group was as follows: 21 per cent had a basic/vocational education, 44 per cent completed a secondary school, and 35 per cent graduated from a college or university. Participation was voluntary with no compensation. All participants were informed about the research and its purposes and gave their informed consent. The study was performed in accordance with the Declaration of Helsinki and approved by the Bioethical Committee (consent no. 255/2020).

### Dependent Variable

We used the Autonomy Preference Index (API) (Ende et al. 1989) to assess respondents’ DA in making healthcare decisions. The API measures two dimensions of patients’ autonomy preference: their desire to make medical decisions and their desire to be informed. The instrument is quick and easy for patients to fill out. The API consists of two subscales: *Information seeking preference and Decision-making preference*. We used all items from *Information seeking preference* subscale (the alpha coefficient reliability for these eight items was 0.7) and four items from the *Decision making preference* subscale (the alpha coefficient reliability for these four items was 0.74) (Table 3 in the appendix). We did not use all the items from the original *Decision-making preference* subscale, because similarly to the studies by other authors (Cullati et al. 2011), not all the items performed expected psychometric criteria and, therefore, we decided not to use them in this analysis. Thus, in our study, DA was determined by adding the scores of two subscales (12 items). The mean and standard deviations for the items used in the analysis are presented in Table 3 in the appendix. Moreover, participants were asked to report their age, gender, and educational level.

### Independent Variable

We used the Polish version of the revised Portrait Values Questionnaire (PVQ-RR) (Schwartz et al. 2012; Cieciuch 2013). The PVQ-R includes 57 short, gender-matched, verbal portraits of different people, each describing a goal important to them. For each portrait, respondents indicate how similar the person is to themselves on a 6-point scale: 1 = not like me at all, 2 = not like me, 3 = a little like me, 4 = moderately like me, 5 = like me, and 6 = very much like me. Respondents’ own values are inferred from the values of the people they describe as similar to themselves. For example, respondents who indicated that a person described by “It is important to him to maintain traditional values or beliefs” is similar to them presumably attribute importance to Tradition.

### Statistical Analysis

Statistical analysis of the results was performed using SPSS statistical software, version 24. The t-test was employed to compare both groups’ means of DA and values. In order to verify the second hypothesis, a regression analysis was conducted with DA as the dependent variable. Because the purpose of the study was to examine whether DA is motivated by the same factors among younger and older adults, we performed the regression analysis separately for the two groups. Relevant results are presented in Table 1 and 2. We considered results to be statistically significant at *p* < .05.

**Table 1.** *Differences in values between younger and older adults. N=421*

Value	Participants	*M*	*SD*	*t-value*	*p-value*
*Achievement*	Younger adults	14.35	2.62	7.65	.001
	Older adults	12.23	3.02		
*Hedonism*	Younger adults	14.18	2.76	4.87	.001
	Older adults	12.80	3.06		
*Stimulation*	Younger adults	11.74	3.48	5.85	.001
	Older adults	9.78	3.39		
*Self-direction-action*	Younger adults	15.79	2.03	4.88	.001
	Older adults	14.66	2.66		
*Self-direction-thought*	Younger adults	15.31	2.17	3.77	.001
	Older adults	14.42	2.58		
*Universalism-tolerance*	Younger adults	14.40	3.07	1.56	*
	Older adults	13.93	3.01		
*Universalism-nature*	Younger adults	12.91	3.55	-3.71	.001
	Older adults	14.11	3.09		
*Universalism-societal concern*	Younger adults	14.06	3.24	-2.17	.05
	Older adults	14.73	3.02		
*Benevolence-caring*	Younger adults	15.59	2.14	-.87	*
	Older adults	15.76	2.04		
*Benevolence-dependability*	Younger adults	15.82	2.26	-.52	*
	Older adults	15.94	2.33		
*Humility*	Younger adults	11.61	2.89	-3.43	.001
	Older adults	12.60	3.0		
*Conformity-interpersonal*	Younger adults	11.40	3.65	-5.11	.001
	Older adults	13.13	3.26		
*Conformity-rules*	Younger adults	10.62	3.35	-9.07	.001
	Older adults	13.50	3.14		
*Tradition*	Younger adults	10.87	4.01	-9.27	.001
	Older adults	14.09	3.08		
*Security-societal*	Younger adults	13.91	3.16	-5.87	.001
	Older adults	15.56	2.49		
*Security-personal*	Younger adults	13.80	2.41	-5.80	.001
	Older adults	15.18	2.48		
*Face*	Younger adults	13.55	2.96	-2.43	.01
	Older adults	14.23	2.74		
*Power-resources*	Younger adults	9.87	3.66	1.11	*
	Older adults	9.48	3.61		
*Power-dominance*	Younger adults	7.91	3.23	3.16	.05
	Older adults	6.93	3.09		

*not significant

**Table 2.** *The results of regression analysis in the group of older (n=199) and younger (n=222) adults*

Value	Younger adults			Older adults		
	*β*	*t-value*	*p-value*	*β*	*t-value*	*p-value*
*Achievement*	-.114	-1.283	*	-.105	-.961	*.
*Hedonism*	-.095	-1.114	*	.088	.824	*.
*Stimulation*	-.025	-.298	*.	.131	1.390	*.
*Self-direction-action*	.183	1.942	.05	.103	1.084	*.
*Self-direction-thought*	.108	1.176	*.	.208	2.231	.05
*Universalism-tolerance*	-.114	-1.128	*.	.114	1.116	*.
*Universalism-nature*	.069	.961	*.	-.060	-.686	*.
*Universalism-societal concern*	-.005	-.048	*.	.091	.820	*.
*Benevolence-caring*	.043	.471	*.	.058	.561	*.
*Benevolence-dependability*	.092	.986	*.	-.144	-1.221	*.
*Humility*	.046	.558	*.	-.288	-3.061	.01
*Conformity-interpersonal*	-.088	-1.057	*.	-.123	-1.207	*.
*Conformity-rules*	-.002	-.029	*.	.140	1.491	*.
*Tradition*	-.151	-1.928	.05	.092	1.056	*.
*Security-societal*	.092	1.174	*.	-.071	-.737	*.
*Security-personal*	-.047	-.516	*.	-.068	-.708	*.
*Face*	.233	2.769	.01	-.053	-.574	*.
*Power-resources*	.102	.949	*.	-.060	-.527	*.
*Power-dominance*	-.167	-1.624	*.	-.137	-1.287	*.

*not significant

## Results

Differences in values between younger and older adults are presented in Table 1. As hypothesized, DA was higher (*t*(420)= 3.43; *p*= .001) in the group of younger adults (*M* = 43.85; *SD* = 4.99) as compared to the group of older adults (*M* = 42.18; *SD* = 4.98). With regard to values, we hypothesized that participants from both groups who attribute high importance to Self-direction would have higher DA. The presumed associations between Self-direction and DA were partially supported by our findings. The results regarding the relationship between DA and values are presented in Table 2. Self-direction-thought was a significant predictor of DA in older adults, whereas Self-direction-action was a significant predictor of DA in younger adults. Thus, the higher Self-direction-thought in older and Self-direction-action in younger adults, the greater the DA. Furthermore, we observed a negative correlation between DA and Humility in older adults, while in younger adults we noticed a positive correlation between DA and Face, and a negative correlation between DA and Tradition. Thus, among younger adults an increase in DA corresponds with an increase in Self-direction-action and Face and a decrease in Tradition, whereas among older adults with an increase in Self-direction-thought and a decrease in Humility. Both regression models turned out to be well adjusted to the variables: younger adults *F*(19, 202) = 2.31; *p* = .002; older adults *F*(19, 178) = 2.03; *p* = .009. Overall, the variables accounted for 10 per cent of the variation in the DA of the younger adults and 9 per cent in the DA of the older adults.

## Discussion

The purpose of this study was to determine the relationship between DA and basic personal values drawn on Schwartz’s value theory. Our results indicate slightly different motivations behind DA among younger and older adults. In the group of younger adults, DA was associated with the desire to be appreciated as a person, motivation to act independently, and the abandonment of traditional order and values, whereas in the group of older adults, DA was associated with independent thinking and a lack of humility.

We confirmed our hypothesis that DA is stronger in the group of younger adults. In Western culture, relying entirely on the physicians to make decisions on his behalf is a remnant of the past. More and more patients prefer sharing decisions with their physicians (Chewning et al. 2012). Naturally, young people are driving this paradigm shift. The abandonment of traditional values is an inherent characteristic of young people, a driving force behind the development of culture and civilization. The cultural background of young people as compared to that of their parents and grandparents is more individualistic, less often devoted to the welfare of others, more focused on self-development, self-protection and self-determination, and therefore also on making autonomous decisions with regard to healthcare. Although previous studies have also revealed a stronger DA in younger people (Levinson et al. 2005; Schneider et al. 2006), our study provided insights into the possible motives behind them.

Our results demonstrate that DA is stronger in people who value Schwartz’s Self-direction. We presumed that in both groups, DA would be related to Self-direction in thinking and acting. It turned out that Self-direction in acting was a significant predictor of DA in the group of younger adults, whereas Self-direction in thinking in the group of older adults. These two aspects of Self-direction represent similar, though slightly different, motivations. Previous studies found that Self-direction–thought items were located nearer Universalism, and Self-direction–action items were located closer Stimulation (Schwartz et al. 2012). According to Schwartz, splitting Self-direction into two more narrowly defined values is valid. “These two subtypes differ in emphases on freedom to cultivate one’s ideas and abilities versus to act as one wishes” (Schwartz et al. 2012, 666). Self-direction–thought refers to developing and using one’s understanding and intellectual competence, whereas Self-direction–action refers to exercising one’s capacity to attain self-chosen goals. Our results confirm the difference in terms of motivation between Self-direction–thought and Self-direction–action. We found that being independent in judgments promoted DA in the group of older adults. Self-direction–thought reflects a value for making one’s own decisions based on one’s own efforts to gather and interpret information. It suggests that in the group of older adults, creative, curious, and independent individuals seem to be more involved in acquiring information and participating in the health decision-making process than those who place less emphasis on these values. A negative correlation between Humility and DA in the group of older adults confirms that in this age group DA is motivated by their willingness to make independent judgments. Humility values emphasize avoiding self-promotion and being satisfied with what one has. Schwartz’s theory assumed that value types situated close to each other on the circle should be positively correlated and the ones situated on the opposite side of the circle—negatively correlated (Cieciuch and Schwartz 2012). This was also the case in our study. Humility, situated on the opposite side of Self-direction, was negatively correlated with DA. Although Schwartz does not define the obverse of Humility, we can assume that giving less importance to Humility may reflect either self-concern or non-compliance with social expectations. Because the essence of Humility is to accept one’s lot (Schwartz et al. 2012), it is very likely that striving for more will be the essence of non-humility. Because people who attribute low importance to Humility are not submissive and obedient, they make up the group of patients who actively negotiate treatment recommendations and question their physicians’ decisions. Such people tend to be assertive, balky, disobedient, insubordinate, defiant, rebellious, contumacious, and conceited. We can conclude that the lack of humbleness and the tendency to think independently led the group of older adults to prefer autonomy.

In the group of younger adults the profile of a person with a strong desire for autonomy was similar, though slightly different. Young people, who attribute high importance to exploring and choosing their own goals, and to relying on themselves, had higher DA. What was the driving force behind DA in this particular age group was the fact that they appreciated the possibility of behaving in line with one’s own goals and views. Thus, we can assume that what favours DA in the group of older adults is their willingness to make independent judgments and in younger adults DA is encouraged by their willingness to behave in accordance with one’s beliefs. Moreover, DA in younger adults was associated with Face. This finding was unexpected. In this age group, the desire to be well informed and the inclination to make independent decisions regarding one’s health was related to the need to protect status and prestige. DA was stronger in younger adults who value the feeling of self-respect and personal worth. It is possible that participating in the decision-making process helps young people feel as equal partners with physicians. This, in turn, enables them to maintain their public image. The relationship between Face and DA was noticed only in the group of young adults. The reason remains unknown. Perhaps it is the result of (1) the specific life situation of young adults and/or (2) their little experience of being ill. Younger adults, at the beginning of their professional careers, want to be appreciated, feel important and be treated as equal partners. Their need to protect their public image and avoid humiliation may be satisfied if they are informed and involved in the decision-making process. Older adults, who value Face, have already had more opportunities to satisfy this value. Moreover, older adults are more likely to have experience in health problems and, therefore, for them participating in decision-making is more about responsibility than maintaining and protecting status and prestige. For older adults, DA is more about solving real problems. Thanks to their experience, they know that participating in decision-making is difficult, and many of them are afraid to take on such a responsibility (Fraenkel and Peters 2009).

In the group of younger adults, a strong desire to be informed and preferences for making medical decisions were associated with a lower level of importance attributed to Tradition. Although it has already been reported that younger adults are generally less attached to a long-established and inherited way of thinking or acting (Schwartz et al. 2012), our results indicate that the reluctance to accept the traditional order may be one of the factors giving rise to DA in this age group. According to Schwartz, Tradition emphasizes maintaining cultural, family, or religious practice. The paternalistic model of patient-doctor relationships can be considered as a traditional one. We found that among young people, those who tend to be more rebellious, anti-authoritarian, and reluctant to accept social norms and customs were more prone to decide on their treatment. Thus, we can assume that the tendency to maintain independence, to protect status and to break with tradition strengthens DA in the group of younger adults.

### Limitations

Our study has several limitations. Firstly, its results are based on a small convenience sample. Participants were university students and their family members. Both groups included a lot of college and university graduates, which limited the generalizability of study results.

Another possible limitation is that the study was conducted as a regional survey and its results may not be generalizable to other geographic settings and populations. In 1989, Poland underwent a political transformation from communism to democracy. Society and its values have significantly changed in the last decades, including an increase of individualism, westernization, and liberalization of lifestyle (Bardi and Schwartz 1996; Schwartz and Bardi 1997). Therefore, the structure of values and their relation to DA may be specific for Poland. In Poland, life expectancy remains below the OECD average (OECD 2021) and health systems are struggling to meet the demands of society. The need to be informed by doctors in this country is not being satisfied (Libura 2016). In the Health at a Glance 2021 report (OECD 2021) Poland ranked last among twenty-one countries in the categories of physician provision of easy-to-understand information and patient involvement in health decisions.

Another potential limitation is that the study was based on a questionnaire. It is not clear whether we would obtain the same results by analysing real-life decisions. Moreover, DA depends on the person’s health status. After receiving a serious diagnosis, people are more willing to take the responsibility of being involved in the decision-making process (Chewning et al. 2012), whereas values influence behaviour more strongly when the pressures from the situation is weaker (Bardi and Schwartz 1996).

Finally, the current research focused on two components of DA: information seeking preference and decision-making preference. Future studies will benefit from investigating the relationship between values and other ways of understanding DA, such as maintaining patient confidentiality and discussing treatment options. Such research will increase our understanding of the complicated motivational mechanisms behind DA. It is, therefore, important to be cautious not to make overgeneralizations from these findings.

## Conclusions

Despite its limitations, our study provided valuable insights into the axiological ground that drive patient preference for autonomy in making medical decisions. The obtained results suggested a variety of motivations behind DA. Our findings revealed that DA understood as a desire to be informed and make own healthcare decisions may result from slightly different reasons in people of particular age groups. Although greater DA in healthcare decisions was associated with a *need for independence* in both young and older adults, the additional reason which tends to favour DA among younger adults were the need to be acknowledged as equal partners in the doctor–patient interaction and the desire to contest the customs and ideas established in traditional culture, whereas lack of humbleness promoted DA among older adults.

The results of our study may be useful in medical practice. The better understanding of the patient’s perspective, the easier to achieve a therapeutic alliance, which in turn promotes a better patient therapeutic care outcome. The current widely accepted approach based on the Calgary-Cambridge model (Kurtz, Silverman, and Draper 2013; Elwyn et al. 2012) recommends taking into account the patient’s perspective, which includes their values ​​when making medical decisions. A doctor incorporating patients’ values can facilitate building the doctor–patient relationship, and thus the message sent by the doctor may be more effective. In the light of our research results, a young patient’s attempts to act independently or question traditional attitudes signal to the doctor that such a patient will want to take a very active part in the decision-making process. This type of young patient will more often choose the independent analysis of written medical information, opting for the informative model of the relationship with the doctors. Appreciating the activity and independence of a young patient will facilitate a better therapeutic understanding. But in the case of elderly patients, a physician can assume a higher level of desire for autonomy by noticing a tendency to contest. It would not be recommendable to encourage them for seeking independent solutions. Such a patient may show a greater need to discuss the information provided by medical professionals, express concern, criticize, put in doubt, ask questions, and he will want to get a picture of the situation through a discursive conversation with the doctor even after obtaining information in writing. These insights might be helpful for medical professionals in building a relationship with patients and for developing accurate communication patterns with different age groups of patients.

## Appendix

Table 3

**Table 3.** *The mean and standard deviations for the items used in the analysis N=421*

Items	*M (SD)*
**Information seeking preference subscale** *:*	
*As you become sicker you should be told more and more about your illness.*	4.49 (0.79)
*You should understand completely what is happening inside your body as a result of your illness.*	4.60 (0.76)
*Even if the news is bad, you should be well informed.*	4.61 (0.76)
*Your doctor should explain the purpose of your laboratory tests.*	4.53 (0.82)
*You should be given information only when you ask for it. R*	2.28 (1.30)
*It is important for you to know all the side effects of your medication.*	3.73 (1.29)
*Information about your illness is as important to you as treatment.*	4.21 (0.96)
*When there is more than one method to treat a problem, you should be told about each one.*	4.68 (0.63)
*As you become sicker you should be told more and more about your illness.*	4.49 (0.79)
*You should understand completely what is happening inside your body as a result of your illness.*	4.60 (0.76)
*Even if the news is bad, you should be well informed.*	4.61 (0.76)
**Decision making preference subscale**:	
*The important medical decisions should be made by the doctor, not by you R*	3.50 (1.31)
*You should go along with your doctor’s advice even if you disagree with it R*	3.75 (1.03)
*When hospitalized, you should not be making decisions about your own care R*	3.99 (1.23)
*If you were sick, as your illness became worse you would want your doctor to take greater control R*	4.28 (0.89)

## References

[CR1] Bardi A, Schwartz SH (1996). Relations among sociopolitical values in Eastern Europe: Effects of the communist experience?. Political Psychology.

[CR2] Beauchamp TL, Childress JF (2019). *Principles of biomedical ethics*.

[CR3] British Medical Journal (1996). Nuremberg Code [1947]. British Medical Journal.

[CR4] Cataldi JR, Sevick C, Pyrzanowski J (2019). Addressing personal parental values in decisions about childhood vaccination: Measure development. Vaccine.

[CR5] Chewning B, Bylund CL, Shah B (2012). Patient preferences for shared decisions : A systematic review. Patient Education and Counseling.

[CR6] Cieciuch J (2013). Pomiar Wartości w Zmodyfikowanym Modelu Shaloma Schwartza [Value measurement in the modified Shalom Schwartz model]. Psychologia Społeczna.

[CR7] Cieciuch, J., and Sh. H. Schwartz. 2012. The number of distinct basic values and their structure assessed by PVQ-40. *Journ*. 10.1080/00223891.2012.655817. 10.1080/00223891.2012.655817 22329443

[CR8] Cullati S, Courvoisier DS, Charvet-Bérard AI, Perneger TV (2011). Desire for autonomy in health care decisions: A general population survey. Patient Education and Counseling.

[CR9] Dworkin G (2015). The nature of autonomy. Nordic Journal of Studies in Educational Policy.

[CR10] Elwyn G, Frosch D, Thomson R (2012). Shared decision making: A model for clinical practice. Journal of General Internal Medcine.

[CR11] Ende J, Kazis L, Ash A, Moskowitz MA (1989). Measuring patients’ desire for autonomy—Decision making and information-seeking preferences among medical patients. Journal of General Internal Medicine.

[CR12] Entwistle VA, Carter SM, Cribb A, McCaffery K (2010). Supporting patient autonomy: The importance of clinician–patient relationships. Journal of General Internal Medicine.

[CR13] Flynn KE, Smith MA (2007). Personality and health care decision-making style. The Journals of Gerontology.

[CR14] Fraenkel L, Peters E (2009). Patient responsibility for medical decision making and risky treatment options. Arthritis Care and Research.

[CR15] Lee SC, Lee SC (2007). On relational autonomy. *The family, medical decision-making, and biotechnology*.

[CR16] Lee YY, Lin JL (2010). Do patient autonomy preferences matter? Linking patient-centered care to patient-physician relationships and health outcomes. Social Science and Medicine.

[CR17] Leppert W, Majkowicz M, Forycka M (2013). Attitudes of Polish physicians and medical students toward breaking bad news, euthanasia and morphine administration in cancer patients. Journal of Cancer Education.

[CR18] Levinson W, Kao A, Kuby A, Thisted RA (2005). Not all patients want to participate in decision making: A national study of public preferences. Journal of General Internal Medicine.

[CR19] Libura, M., E. Borek, K. Maciorowska, J. Turkiewicz, and A. Sitek. Lekarze i pacjenci w Polsce—model relacji w gabinecie lekarskim AD 2016 [Doctors and patients in Poland—the relationship model in a doctor's office: 2016]. https://mypacjenci.org/wp-content/uploads/2018/08/Empowerment_RAPORT.pdf. Accessed June 27, 2022.

[CR20] Kurtz S, Silverman J, Draper J (2013). *Skills for communicating with patients*.

[CR21] Loge JH, Kaasa S, Ekeberg Ø, Falkum E, Hytten K (1996). Attitudes toward informing the cancer patient—A survey of Norwegian physicians. European Journal of Cancer Part A.

[CR22] Marcus J, Ceylan S, Ergin C (2017). Not so “traditional” anymore? Generational shifts on Schwartz values in Turkey. Journal of Cross-Cultural Psychology.

[CR23] Mfutso-Bengo J, Masiye F, Myser C (2011). Toward an African Ubuntuology/Umunthuology bioethics in Malawi in the context of globalization. *Bioethics around the globe*.

[CR24] Naczelna Izba Lekarska. 2004. The Polish Medical Code of Ethics*. Announcement No. 1/04/IV of the President of the Supreme Medical Council*. https://nil.org.pl/dokumenty/kodeks-etyki-lekarskiej. Accessed June 27 2022.

[CR25] National Commission for the Protection of Human Subjects of Biomedical and Behavioral Research. 1978. Belmont Report: Ethical principles and guidelines for the protection of human subjects of research. 25951677

[CR26] OECD. 2021. Health at a glance 2021. https://www.oecd-ilibrary.org/social-issues-migration-health/health-at-a-glance-2021_ae3016b9-en. Accessed June 28, 2022.

[CR27] Onuoha, Ch. 2007. Bioethics across borders. An African perspective. *ACTA UNIVERSITATIS UPSALIENSIS Uppsala Studies in Social Ethics 34*. PhD dissertation, Uppsala: Uppsala University.

[CR28] Robinson OC (2013). Values and adult age: Findings from two cohorts of the European social survey. European Journal of Ageing.

[CR29] Roccas S, Sagiv L, Schwartz SH, Knafo A (2002). The big five personality factors and personal values. Personality and Social Psychology Bulletin.

[CR30] Sagiv L, Sverdlik N, Schwarz N (2011). To compete or to cooperate? Values’ impact on perception and action in social dilemma games. European Journal of Social Psychology.

[CR31] Schneider A, Körner T, Mehring M, Wensing M, Elwyn G, Szecsenyi J (2006). Impact of age, health locus of control and psychological co-morbidity on patients’ preferences for shared decision making in general practice. Patient Education and Counseling.

[CR32] Schwartz SH (1994). Are there universal aspects in the structure and contents of human values?. Journal of Social Issues.

[CR33] Schwartz SH (2012). An overview of the Schwartz theory of basic values. Online Readings in Psychology and Culture.

[CR34] Schwartz SH, Bardi A (1997). Influences of adaptation to communist rule on value priorities in Eastern Europe. Political Psychology.

[CR35] Schwartz SH, Cieciuch J, Vecchione M (2012). Refining the theory of basic individual values. Journal of Personality and Social Psychology.

[CR36] Schwartz SH, Cieciuch J, Vecchione M, Torres C, Dirilen-Gumus O, Butenko T (2017). Value tradeoffs propel and inhibit behavior: Validating the 19 refined values in four countries. European Journal of Social Psychology.

[CR37] Skimina E, Cieciuch J, Schwartz SH, Davidov E, Algesheimer R (2019). Behavioral signatures of values in everyday behavior in retrospective and real-time self-reports. Frontiers in Psychology.

[CR38] Tao J, Po-Wah L, Tao J, Po-Wah L (2002). Global bioethics, global dialog: Introduction. *Cross-cultural perspectives on the (im)possibility of global bioethics*.

[CR39] Varelius J (2006). The value of autonomy in medical ethics. Medicine, Health Care and Philosophy.

[CR40] Veatch RM, Hoshino K (1997). Autonomy and communitarianism: The ethics of terminal care in cross-cultural perspective. *Japanese and Western Bioethics. Philosophy and Medicine, Vol 54*.

[CR41] Anon (2017). World Medical Association Declaration of Geneva. African Health Science.

[CR42] World Medical Association. 1964. Declaration of Helsinki, formal statement of ethical principles published by the World Medical Association (WMA) to guide the protection of human participants in medical research.

[CR43] Yu X, Li W (2014). Informed consent and ethical review in Chinese human experimentation: Reflections on the “Golden Rice Event”. Biotechnology Law Report.

